# Association between living alone and incident type 2 diabetes among middle-aged individuals in Korea: a nationwide cohort study

**DOI:** 10.1038/s41598-021-82868-z

**Published:** 2021-02-11

**Authors:** Ga Eun Nam, Wonsock Kim, Kyungdo Han, Jin-Hyung Jung, Byoungduck Han, Jinwook Kim, Nan Hee Kim, Kyung Mook Choi, Kyung Hwan Cho, Yong Gyu Park, Seon Mee Kim

**Affiliations:** 1 Department of Family Medicine, Korea University College of Medicine, Seoul, Republic of Korea; 2 Department of Statistics and Actuarial Science, Soongsil University, Seoul, Republic of Korea; 3 Department of Biostatistics, Catholic University of Korea, Seoul, Republic of Korea; 4 Division of Endocrinology and Metabolism, Department of Internal Medicine, Korea University College of Medicine, Seoul, Republic of Korea

**Keywords:** Endocrinology, Health care

## Abstract

We studied the association between living alone and the risk of incident type 2 diabetes in middle-aged individuals using nationwide cohort data from the Korean population. 11,686, 677 middle-aged individuals aged 40–64 years who underwent health examinations by the Korean National Health Insurance System between 2009 and 2012 were followed up until December 31, 2015. The hazard ratios (HRs) and 95% confidence intervals (CIs) were estimated using multivariable Cox proportional hazards regression analysis. During the median follow-up duration of 5.6 years, 393,438 individuals developed type 2 diabetes. Living alone was significantly associated with incident type 2 diabetes in all adjusted models (HR 1.08; 95% CI 1.07–1.09 in model 4). Individuals who lived alone for < 1 year and 1–7 years were associated with increased HRs of 1.07 (1.04–1.09) and 1.08 (1.07–1.09). Living alone was associated with incident type 2 diabetes in all subgroups. The association was stronger in men than in women and younger individuals than in older individuals. Living alone, even for a short duration, may be an important factor in type 2 diabetes development. Better household conditions and appropriate support to one-person households may be needed to prevent type 2 diabetes.

## Introduction

Living arrangements have changed remarkably in recent decades. In particular, one-person households are consistently increasing worldwide; approximately 30% of individuals in Western countries live alone, and the proportion of middle-aged individuals has markedly increased^1,2^. Moreover, the proportion of one-person households in Korea has dramatically increased from 7% in 1985 to 24% in 2010^3^. Accordingly, there have been concerns that living alone may influence lifestyle factors and health conditions, including the development of type 2 diabetes^4–6^.

Type 2 diabetes is a common metabolic disease that causes several complications, including microvascular and cardiovascular diseases and mortality^7^. Globally, 425 million adults were affected by diabetes in 2015, and the number is expected to reach 642 million by 2040^8^. The prevalence of type 2 diabetes in Korea has also dramatically increased from ≤ 1% in 1960 to 13.7% in 2014^9^, and the prevalence is significantly observed in younger individuals, including middle-aged individuals^10^. Diabetes and diabetes-related complications increase the healthcare burden on individuals and healthcare systems, including medical costs^11,12^, and the prevention of type 2 diabetes has become a major public health issue worldwide^8^. Moreover, younger individuals with early onset type 2 diabetes are likely to be more obese and have poorer glycemic control and higher lipid levels than older individuals^13,14^.

While several lifestyle factors, such as high calorie and saturated fatty acid intake, physical inactivity, alcohol consumption, and smoking, are recognized as modifiable risk factors for type 2 diabetes^15–17^, the association between household conditions, such as living alone, and type 2 diabetes has been scarcely studied. As previously mentioned, lifestyle factors related to type 2 diabetes may be influenced by societal conditions, including housing pattern^4,5^. Moreover, only a few studies in Western countries have examined the association between living arrangements and type 2 diabetes. In a German cohort study of individuals living alone, men were more likely to develop type 2 diabetes than women^6^. A Swedish study of 461 middle-aged women with impaired glucose tolerance (IGT) reported that women living alone were at risk of developing type 2 diabetes and suggested living alone as a possible predictive factor for incident type 2 diabetes^18^. Although socioeconomic and cultural backgrounds and the nature of type 2 diabetes are significantly different between nations or ethnicities, the associations between living arrangements and type 2 diabetes in Asian nations have been poorly studied. Therefore, we investigated the association between living alone and incident type 2 diabetes in middle-aged individuals using nationwide cohort data from the Korean population.

## Methods

### Data sources and study population

This study was based on data provided by the Korean National Health Insurance Corporation (NHIC). Since 2000, the Korean NHIC has managed the National Health Insurance System (NHIS), a single universal insurance system. The Korean NHIS recommends biannual health examinations for all insured individuals. Therefore, it stores extensive health information of nearly the entire Korean population, including data on demographics, disease diagnosis based on the International Classification of Diseases, 10th Revision, Clinical Modification (ICD-10-CM), medical treatment and procedures, and health examination results. Since 2015, the database has been released to researchers whose study protocol has been approved by the official review committee.

**Figure 1:**
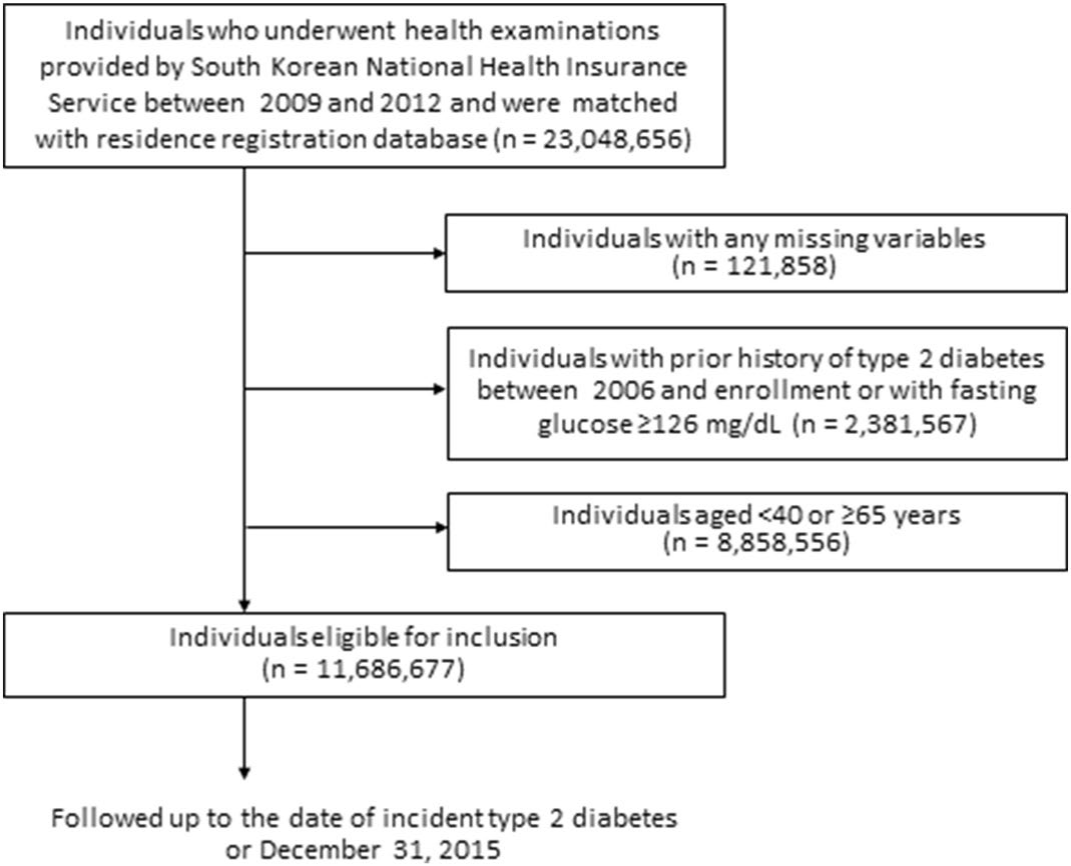
Flow chart of study population.

In this study, we initially considered 23,048,658 individuals who underwent a health examination by the Korean NHIS between January 1, 2009, and December 31, 2012 and their NHIS registration database was matched with their residence registration database. Among them, we excluded individuals with missing variables (n = 121,858), those who had been diagnosed with type 2 diabetes based on the ICD-10-CM code (E11-14) between 2006 and upon enrollment in this study, or who had fasting blood glucose levels ≥ 126 mg/dL during health examination upon enrollment in this study (n = 2,381,567), and those aged < 40 years or ≥ 65 years (n = 8,858,556). Finally, 11,686,677 middle-aged individuals were included and followed-up until December 31, 2015. The median follow-up duration was 5.6 years (interquartile range 4.6–6.2 years). The flow chart of the study population is shown in Fig. 1. This study adhered to the principles of the Declaration of Helsinki and was approved by the Institutional Review Board of Korea University Guro Hospital (number: 2017GR0290).

### Definition of one-person household

We matched the participants’ NHIS registration and residence registration databases every year. We defined one-person households as individuals who were householders and did not have any other household members whom they could match their NHIS database with their residence registration database. The duration of living alone was defined by the total number of years the participants lived in one-person households prior to enrollment. We assessed whether the participants had lived in one-person households or not every year before enrollment by matching the NHIS database and residence registration database and adding up the total number of years that participants lived in one-person households. We then categorized the duration of living alone as < 1 or 1–7 years.

### Study outcome

The study outcome was newly diagnosed type 2 diabetes. A diagnosis of type 2 diabetes was established when the fasting blood glucose level was ≥ 126 mg/dL or when claims for disease diagnosis and antidiabetic medication prescription were established along with the ICD-10-CM code of E11-14.

### Covariates

Participants’ demographic and lifestyle characteristics were collected using a standardized self-reports questionnaire. Income level was divided into the lower 20% and the other groups. Residential areas were classified as metropolitan cities and other areas. According to the classification of administrative districts in Korea, metropolitan cities are cities that reflect residential areas with a population of more than one million and include Seoul, Busan, Incheon, Daegu, Daejeon, Gwangju, and Ulsan. Smoking status was classified as non-smoker, former smoker, or current smoker. Individuals who consumed ≥ 30 g of alcohol per day were considered heavy alcohol drinkers^19^. Regular physical exercise was defined as individuals who performed moderate-intensity exercise for ≥ 5 days per week or high-intensity exercise ≥ 3 days per week. The NHIS also provided results of health examinations, such as anthropometric measurements and blood chemistry. The participants body weight, height, and waist circumference were measured, and body mass index (BMI) was calculated as weight divided by squared height. We defined BMI ≥ 25 kg/m^2^ as obesity^20^. Using a standardized sphygmomanometer, blood pressure was measured in a sitting position after at least 5 min of rest. Blood samples were obtained after overnight fasting to measure the serum levels of glucose, total cholesterol, triglycerides, high-density lipoprotein cholesterol, and low-density lipoprotein cholesterol (LDL-C). Participants’ past medical history, including hypertension and dyslipidemia, was defined based on health examination results and claims for disease diagnosis and medication prescription along with the ICD-10-CM code. Underlying hypertension was defined as blood pressure ≥ 140/90 mmHg or disease diagnosis and claims for antihypertensive medication under the ICD-10-CM codes of I10-13 and I15. Dyslipidemia was defined as serum total cholesterol level ≥ 240 mg/dL or disease diagnosis and claims for lipid-lowering medication under the ICD-10-CM code E78.

### Statistical analysis

Statistical analyses were performed using Statistical Analysis System (SAS) software (version 9.4; SAS institute, Cary, NC, USA). We presented the baseline characteristics of study participants according to living arrangement (living alone and together) as mean ± standard deviation for continuous variables and number (percentage) for categorical variables. The values were compared using an independent t-test or chi-squared test. We calculated the incidence rate of type 2 diabetes by dividing the number of events by 1000 person-years. We plotted Kaplan–Meier curves regarding the incidence probability of type 2 diabetes according to living arrangements. We performed multivariable Cox hazard regression analysis to determine the association between living arrangement and risk of incident type 2 diabetes and calculated the hazard ratio (HR) and 95% confidence interval (CI). Model 1 was unadjusted, and Model 2 was adjusted for age and sex. We additionally adjusted for smoking status, alcohol consumption, and income in Model 3. In Model 4, we further adjusted for BMI, hypertension, and dyslipidemia plus variables in model 3. We also examined the associations in several subgroups related to living arrangement and type 2 diabetes, such as age, sex, smoking status, alcohol consumption, physical activity, income, obesity, hypertension, and dyslipidemia and calculated the *P* value for interaction using Cox regression analysis.

## Results

### Baseline characteristics

Table 1 shows the baseline characteristics according to living arrangements. At baseline, 11.3% (n = 1,325,974) of the study participants were living alone, and 393,438 (3.4%) developed type 2 diabetes during the median follow-up duration of 5.6 years. The mean age and proportion of males were higher in individuals living alone than in those living together. The proportion of current smokers and heavy alcohol drinkers was significantly higher in individuals living alone than in those living together. Individuals living alone were likely to have lower incomes and to live in rural areas than those living together. Baseline cardiometabolic parameters, such as waist circumference, blood pressure, and serum levels of fasting glucose, total cholesterol, triglycerides, and LDL-C, were higher in individuals living alone than in those living together. The prevalence of hypertension and dyslipidemia was also significantly higher in one-person households than in multimember households.

**Table 1:** Baseline characteristics of study participants according to living arrangement.

	Living together	Living alone	*P*
N	10,360,703	1,325,974	
Age (years)	49.9 ± 7.0	51.3 ± 7.3	< 0.001
Sex (male)	4,707,674 (45.4)	630,479 (47.6)	< 0.001
**Duration of living alone (years)**			< 0.001
< 1	·	220,023 (16.6)	
1–7	·	177,732 (83.4)	
Current smoker	2,128,018 (20.5)	381,673 (28.8)	< 0.001
Heavy alcohol drinker	752,237 (7.3)	120,802 (9.1)	< 0.001
Regular exerciser	2,000,563 (19.3)	246,948 (18.6)	< 0.001
Income (lower 20%)	2,034,083 (19.6)	500,740 (37.8)	< 0.001
Residential area (metropolitan)	4,873,667 (47.1)	583,077 (44.0)	< 0.001
Height (cm)	162.4 ± 8.4	162.1 ± 8.5	< 0.001
Weight (kg)	63.1 ± 10.6	62.8 ± 10.7	< 0.001
BMI (kg/m^2^)	23.8 ± 3.0	23.8 ± 3.1	< 0.001
WC (cm)	80.0 ± 8.6	80.3 ± 8.8	< 0.001
Systolic BP (mmHg)	121.8 ± 14.8	122.7 ± 15.3	< 0.001
Diastolic BP (mmHg)	76.4 ± 10.2	77.0 ± 10.4	< 0.001
Fasting glucose (mg/dL)	93.7 ± 11.2	94.1 ± 11.5	< 0.001
Total cholesterol (mg/dL)	199.4 ± 36.1	201.4 ± 37.3	< 0.001
Triglycerides (mg/dL)	112.4 (112.3–112.4)	118.3 (118.2–118.4)	< 0.001
HDL-C (mg/dL)	55.9 ± 19.8	56.1 ± 19.2	< 0.001
LDL-C (mg/dL)	118.4 ± 41.1	118.7 ± 41.3	< 0.001
Hypertension	2,531,105 (24.4)	362,855 (27.4)	< 0.001
Dyslipidemia	2,006,990 (19.4)	295,165 (22.3)	< 0.001

*BMI* body mass index, *WC* waist circumference, *BP* blood pressure, *HDL-C* high-density lipoprotein cholesterol, *LDL-C* low-density lipoprotein cholesterol.

Data are presented as mean ± standard deviation or number (%).

### Longitudinal association between living arrangements and incident type 2 diabetes

**Figure 2:**
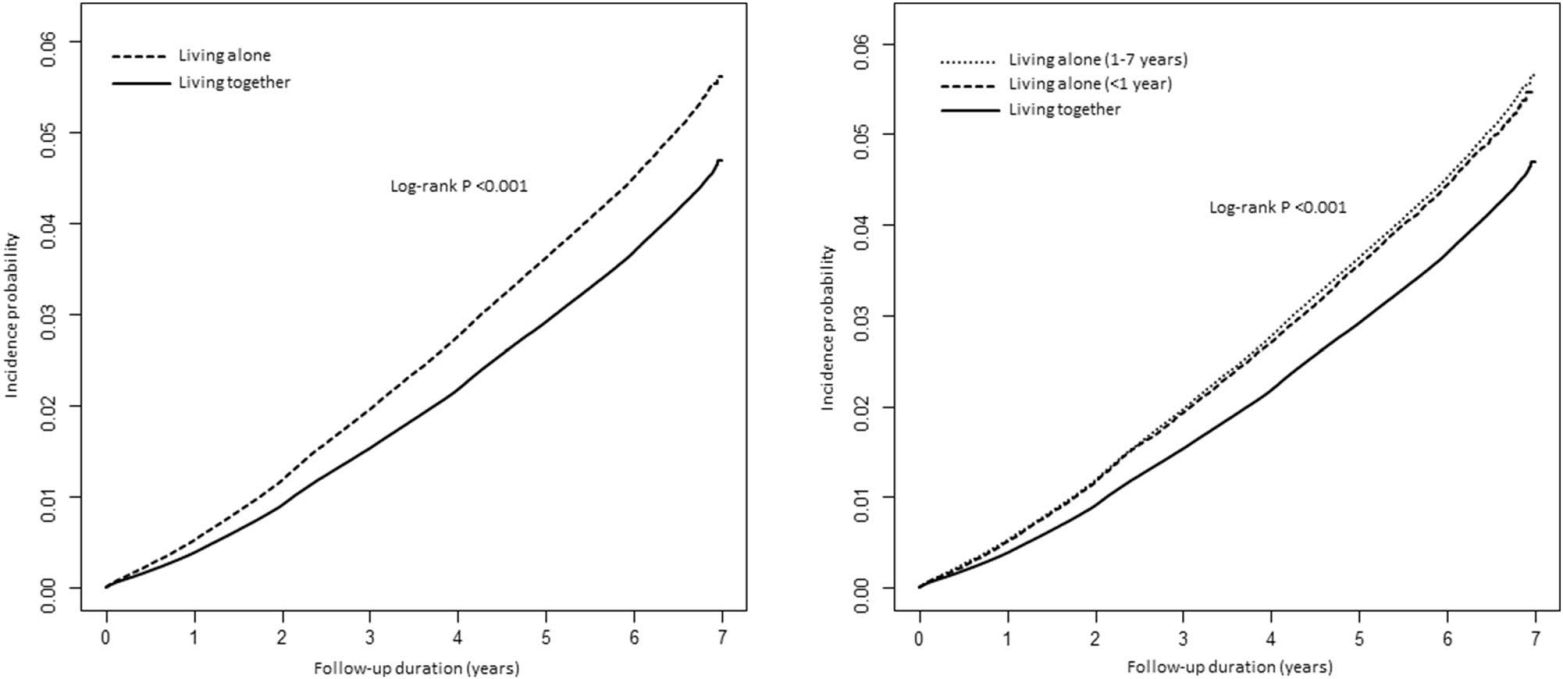
Kaplan–Meier curves for incidence probability of type 2 diabetes according to living arrangement.

Figure 2 shows the incidence probability of type 2 diabetes according to living arrangement. The incidence probability was significantly higher in the living alone group than in the living together group (log-rank *P* < 0.001). It significantly increased from multimember households to one-person household within 1 year and one-person household for 1–7 years (log-rank *P* < 0.001). Table 2 shows the HR (95% CI) of incident type 2 diabetes according to living arrangements. Individuals living alone was significantly associated with an increased risk of incident type 2 diabetes compared to those living together in all adjusted models (HR, 95% CI 1.24, 1.23–1.25 in model 1; 1.14, 1.13–1.15 in model 2; 1.09, 1.08–1.11 in model 3; and 1.08, 1.07–1.09 in model 4). Individuals who lived alone within 1 year and during 1–7 years were associated with increased HR of 1.07 (95% CI 1.04–1.09) and 1.08 (1.07–1.09), respectively, compared to individuals living together.

**Table 2:** Associations between living arrangement and risk of incident type 2 diabetes mellitus.

	N	Events	Person-years	IR^a^	HR (95% CI)			
					Model 1^b^	Model 2^c^	Model 3^d^	Model 4^e^
**Living arrangement**								
Living together	10,360,703	341,341	55,504,775	6.15	1 (ref.)	1 (ref.)	1 (ref.)	1 (ref.)
Living alone	1,325,974	52,097	6,899,120	7.55	1.24 (1.23–1.25)	1.14 (1.13–1.15)	1.09 (1.08–1.11)	1.08 (1.07–1.09)
*P*					< 0.001	< 0.001	< 0.001	< 0.001
**Duration of living alone**								
Living together	10,360,703	341,341	55,504,775	6.15	1 (ref.)	1 (ref.)	1 (ref.)	1 (ref.)
< 1 year	165,856	6607	886,620	7.45	1.21 (1.18–1.24)	1.10 (1.07–1.13)	1.07 (1.05–1.10)	1.07 (1.04–1.09)
1–7 years	1,160,118	45,490	6,012,500	7.57	1.24 (1.23–1.25)	1.15 (1.14–1.16)	1.10 (1.09–1.11)	1.08 (1.07–1.09)
*P*					< 0.001	< 0.001	< 0.001	< 0.001

*IR* incidence rate, *HR* hazard ratio, *CI* confidence interval.

^a^Incidence of type 2 diabetes mellitus per 1000 person-years.

^b^Model 1 was unadjusted.

^c^Model 2 was adjusted for age and sex.

^d^Model 3 was adjusted for age, sex, smoking status, alcohol consumption, physical activity, and income.

^e^Model 4 was adjusted for age, sex, smoking status, alcohol consumption, physical activity, income, body mass index, hypertension, and dyslipidemia.

### Subgroup analyses

**Figure 3:**
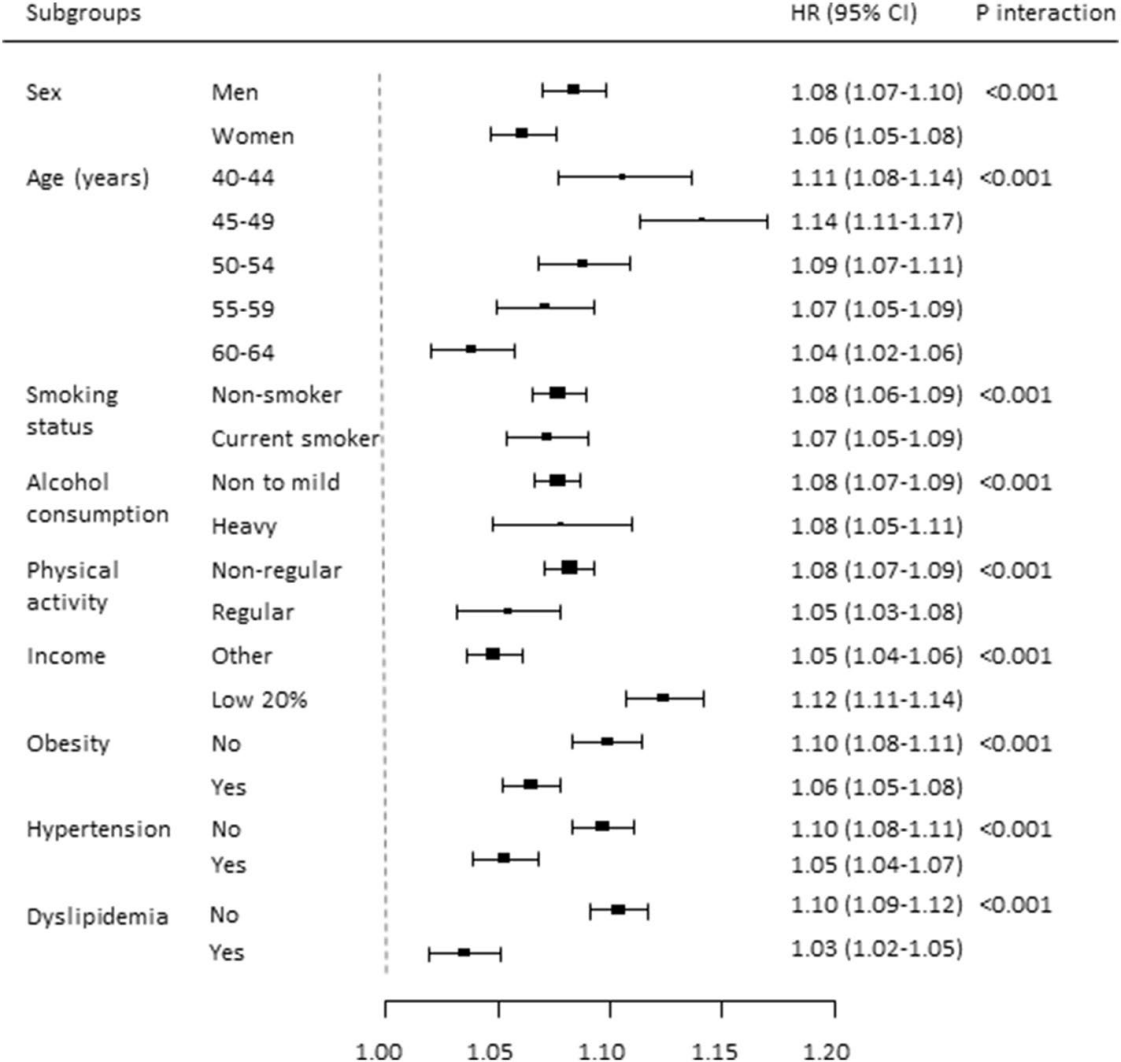
Subgroup analysis regarding the association between living alone and risk of incident type 2 diabetes.

Figure 3 presents the results from subgroup analyses regarding the association between living alone and the risk of type 2 diabetes, with individuals living together as the reference after adjusting for potential confounding variables. Living alone was associated with an increased risk of incident type 2 diabetes in all the subgroups. The association was stronger in men than in women, in younger individuals (40–49 years) than in older individuals (50–64 years), and in the lower income group than in the higher income group. The association was more significant in nonsmokers than in current smokers, in individuals without obesity than in those with obesity, in individuals with hypertension in those without hypertension, and in individuals with dyslipidemia than in those without dyslipidemia (*P* < 0.001 for all interactions).

## Discussion

In this large-scale cohort study of 11.6 million Korean individuals, middle-aged individuals who lived alone were independently associated with an increased risk of type 2 diabetes compared to those who lived together, and showed 8% higher risk of type 2 diabetes even after adjusting for potential confounding variables. Living alone for < 1 year and for 1–7 years was associated with a 7% and 8% increase in the risk of type 2 diabetes, respectively; living alone for 1–7 years had a more significant association with incident type 2 diabetes risk than living alone for < 1 year; however, the risk of type 2 diabetes in individuals who lived alone for < 1 year was comparable to the risk among those who lived alone for 1–7 years. Subgroups of men, younger age (40–49 years), lower income, and those without cardiovascular risk factors such as current smoking, obesity, hypertension, and dyslipidemia, exhibited stronger associations between living alone and type 2 diabetes risk, compared to the other subgroups. Our findings suggest that living alone may be a risk factor for incident type 2 diabetes in middle-aged individuals, with even a short duration of living alone being an important risk factor for type 2 diabetes. One-person households with male sex, younger age, lower income, and metabolically healthy status may be at a higher risk of type 2 diabetes incidence than multimember households.

Few previous studies have examined the association between living arrangements and the risk of incident type 2 diabetes. A German 10-year follow-up cohort study of 8,807 adults aged 35–74 years reported a significantly positive association between living alone and the risk of type 2 diabetes only in men^6^. In this study, the HRs of developing type 2 diabetes in individuals who lived alone compared to those who did not were 1.89 (95% CI 1.33–2.70) in men and 0.83 (95% CI 0.52–1.32) in women after adjusting for education and depressed mood in addition to known risk factors for type 2 diabetes. A Swedish study of 461 women aged 50–64 years with IGT, showed that women living alone had an increased [odds ratio (OR) 3.03] risk of type 2 diabetes, after adjusting for biological risk factors, educational level, occupation, subjective mental health, exercise status, dietary regimen, and alcohol consumption. However, when smoking status was additionally adjusted, the OR was 2.07 (95% CI 0.62–6.88) with attenuated significance^18^. Our study results are partly consistent with previous findings, but we considered a significantly larger sample size comprising middle-aged individuals with several confounding factors, including smoking status. Therefore, our study provides more concrete evidence of the association between living alone and type 2 diabetes. Additionally, our findings may be supported by the previous studies on the association between marital status and health status, including type 2 diabetes. An Australian 3-year follow-up cohort study conducted in elderly women reported that not having a partner and having a mental health index score within the clinically normal range could be predictors of diabetes after multivariable adjustment^21^. Another study found that married individuals were likely to have more social support, better economic conditions, and longer survival than single individuals^22^.

The underlying mechanism of the association between living alone and incident type 2 diabetes is unclear. Living alone is a risk factor for social isolation, which leads to low emotional support and psychosocial stress^23^. Stress may influence the hypothalamus-pituitary-adrenocortical axis function, resulting in increased secretion of glucocorticoids, decreased glucose uptake, and increased circulating glucose levels^24,25^. Stress can also increase sympathetic adrenal system activity, which is associated with increased blood glucose and IGT^26^; stress may lead to a significantly increased risk of type 2 diabetes^27,28^. These processes can be observed in depressive disorder, which is related to the long-term activation of the stress system^29,30^. Individuals living alone are more likely to develop depression due to their poorer economic status than individuals who are not living alone^31^. Living alone is associated with financial, social, lifestyle, and environmental factors, which are likely to influence nutrition behaviors^32,33^. Participants living alone may have poorer and insufficient intakes of some core foods, including fruits, vegetables, and fish, compared to participants not living alone^34^, which may lead to chronic diseases, such as type 2 diabetes.

It is interesting to note that living alone had a stronger association with incident type 2 diabetes in men than in women. Similarly, the aforementioned study reported that the positive association between one-person households and type 2 diabetes was observed only in men; this difference may be due to the difference in the ability to manage food and nutritional needs between the sexes. Some suggested that women may be more confident in managing life than men as a result of the educational system in society^34^. Younger individuals (40–49 years), particularly those aged 45–49 years had a stronger association with type 2 diabetes than older individuals (50–64 years). In Korea, there is a social phenomenon called “kirogi” (or wild goose) families, which are Korean families separated by an ocean. They almost send 50–100% of their family income abroad and form one-person households usually in the early middle-age. This status may have a significant influence on the association between younger age and type 2 diabetes. Moreover, we found that the association was more significant in participants without traditional cardiovascular risk factors, such as obesity, hypertension, and dyslipidemia than in those with traditional cardiovascular risk factors. Healthy individuals need to be carefully monitored if they live alone. Further studies are required.

Our study had several limitations that need to be addressed. First, although we assessed the household condition every year, it was difficult to determine the exact period of living alone between the assessments. Second, depressive mood and marital status could not be adjusted due to insufficient data. Third, since the analytic sample was limited to middle-aged Korean individuals, additional studies in young and elderly individuals and other ethnic groups are required to confirm the generalizability of our results. Despite these limitations, the major strength of our study was its large sample size. To the best of our knowledge, this is the first study to examine the association between living alone and incident type 2 diabetes using nationwide cohort data. This enabled us to perform subgroup analyses and adjust for several confounding variables, including income and smoking status, which are important risk factors in type 2 diabetes^35,36^. A previous study assessed household conditions only once among the participants. In addition, we checked the living arrangement every year during follow-up to minimize misclassification bias in the changes in household condition.

In conclusion, living alone was independently associated with incident type 2 diabetes in middle-aged Korean individuals, with even a short duration of living alone being significantly associated with incident type 2 diabetes. Our findings suggest that living arrangements, such as living alone, may be related to type 2 diabetes in middle-aged East Asians. Consideration of household conditions and appropriate support for one-person households may be useful in the prevention of type 2 diabetes in this group.
